# Dichotomous Organization of the External Globus Pallidus

**DOI:** 10.1016/j.neuron.2012.04.027

**Published:** 2012-06-21

**Authors:** Nicolas Mallet, Benjamin R. Micklem, Pablo Henny, Matthew T. Brown, Claire Williams, J. Paul Bolam, Kouichi C. Nakamura, Peter J. Magill

**Affiliations:** 1 Medical Research Council Anatomical Neuropharmacology Unit, Department of Pharmacology and Oxford Parkinson's Disease Centre, University of Oxford, Oxford OX1 3TH, UK

## Abstract

Different striatal projection neurons are the origin of a dual organization essential for basal ganglia function. We have defined an analogous division of labor in the external globus pallidus (GPe) of Parkinsonian rats, showing that the distinct temporal activities of two populations of GPe neuron in vivo are underpinned by distinct molecular profiles and axonal connectivities. A first population of prototypic GABAergic GPe neurons fire antiphase to subthalamic nucleus (STN) neurons, often express parvalbumin, and target downstream basal ganglia nuclei, including STN. In contrast, a second population (arkypallidal neurons) fire in-phase with STN neurons, express preproenkephalin, and only innervate the striatum. This novel cell type provides the largest extrinsic GABAergic innervation of striatum, targeting both projection neurons and interneurons. We conclude that GPe exhibits several core components of a dichotomous organization as fundamental as that in striatum. Thus, two populations of GPe neuron together orchestrate activities across all basal ganglia nuclei in a cell-type-specific manner.

## Highlights

► Identified key elements of fundamentally-important dichotomous organization of GPe ► First correlation of GPe neuron firing in vivo with neurochemistry and structure ► Discovered arkypallidal neurons that massively and specifically innervate striatum ► Defined new factors for widespread and excessive beta oscillations in Parkinson's disease

## Teaser

By rigorously defining the neurochemical and structural properties of individual neurons recorded in vivo, Mallet et al. reveal the foundations of a striking functional dichotomy in the external globus pallidus, including a novel cell type that exclusively targets striatum.

## Introduction

Functional dichotomy in striatal projection neurons is pivotal for the hugely influential “direct/indirect pathways” model of basal ganglia (BG) organization (Albin et al., 1989; Bergman et al., 1990; Gerfen and Surmeier, 2011; Smith et al., 1998; Wichmann and DeLong, 1996). Two major types of medium-sized densely-spiny neuron (MSN) preferentially innervate either external globus pallidus (GPe) or BG output nuclei (the internal globus pallidus, also known as the entopeduncular nucleus [EPN] in rodents, and the substantia nigra pars reticulata [SNr]). They are further distinguished by distinct electrophysiological properties, selective expression of neuropeptides and dopamine receptors, and their opposing influences on behavior (Gerfen and Surmeier, 2011; Kravitz et al., 2010). Dopamine balances these two striatal outputs, and its loss in Parkinson's disease (PD) promotes functional extremes, with disastrous behavioral consequences (Albin et al., 1989; Wichmann and DeLong, 1996). Such models posit feed-forward information transfer in BG, with striatum “upstream” and subthalamic nucleus (STN) “downstream” of GPe. Although these models and concepts are idealized, such that they do not capture the full gamut of interactions of GPe or other BG nuclei (Bevan et al., 2002; Smith et al., 1998), they provide rationale for many pharmacological and surgical interventions in PD (Bergman et al., 1990; Schapira et al., 2006).

The GPe is an integrative hub for coordinating neuronal activity across the BG (Kita, 2007; Smith et al., 1998) and, in contrast to striatum, is almost always embodied as a homogeneous entity in circuit-level descriptions (Albin et al., 1989; Alexander and Crutcher, 1990; Smith et al., 1998; Wichmann and DeLong, 1996). While different GPe domains engage in diverse functions (Alexander and Crutcher, 1990; Kelly and Strick, 2004; Smith et al., 1998), each is theoretically populated by the same cell type. Accordingly, the notional “prototypic” vertebrate GPe neuron is a fast-firing GABAergic cell that supports uniform function by always innervating STN (Bevan et al., 2002; Smith et al., 1998; Stephenson-Jones et al., 2011), despite GPe cellular heterogeneity being reported at many levels (Flandin et al., 2010; Hoover and Marshall, 2002; Kita, 2007; Kita and Kitai, 1994; Sadek et al., 2007). While physiological diversity exists in normal GPe in vivo (DeLong, 1971; Mallet et al., 2008a), it is exacerbated by dopamine loss, as exemplified in the 6-hydroxydopamine (6-OHDA)-lesioned rat model of PD (Mallet et al., 2008a). Thus, two populations of GPe unit (“GP-TI” and “GP-TA” (Mallet et al., 2008a); acronyms explained below) are readily distinguished by their distinct temporal activities, such as preferentially firing at different phases of the exaggerated beta-frequency (15–30 Hz) oscillations that accompany movement difficulties in PD patients and this animal model (Brown et al., 2001; Hammond et al., 2007; Mallet et al., 2008a, 2008b). Explaining such heterogeneity is imperative for understanding BG function/dysfunction, and it requires correlation of the activity, neurochemistry, and outputs of individual GPe neurons in vivo.

Exploiting the heightened GPe physiological duality in Parkinsonism, we demonstrate that distinct temporal activities correlate with distinct neurochemical and structural properties. Identified GABAergic GP-TI neurons are prototypic and innervate downstream BG nuclei. However, GP-TA neurons are not, because rather than targeting STN, they provide a massive and specific GABAergic/enkephalinergic innervation of striatum. Thus, two GPe cell populations are specialized to fulfill broadly complementary roles in BG circuits, emulating the dichotomous striatal organization. Moreover, our data suggest that any controlling input to GP-TA neurons is, by virtue of the unique properties of this cell type, well positioned to powerfully influence activity along the direct pathway, the indirect pathway, or both of the output pathways of striatum.

## Results

### Physiological Dichotomy of External Globus Pallidus Neurons after Dopamine Loss

**Figure 1:**
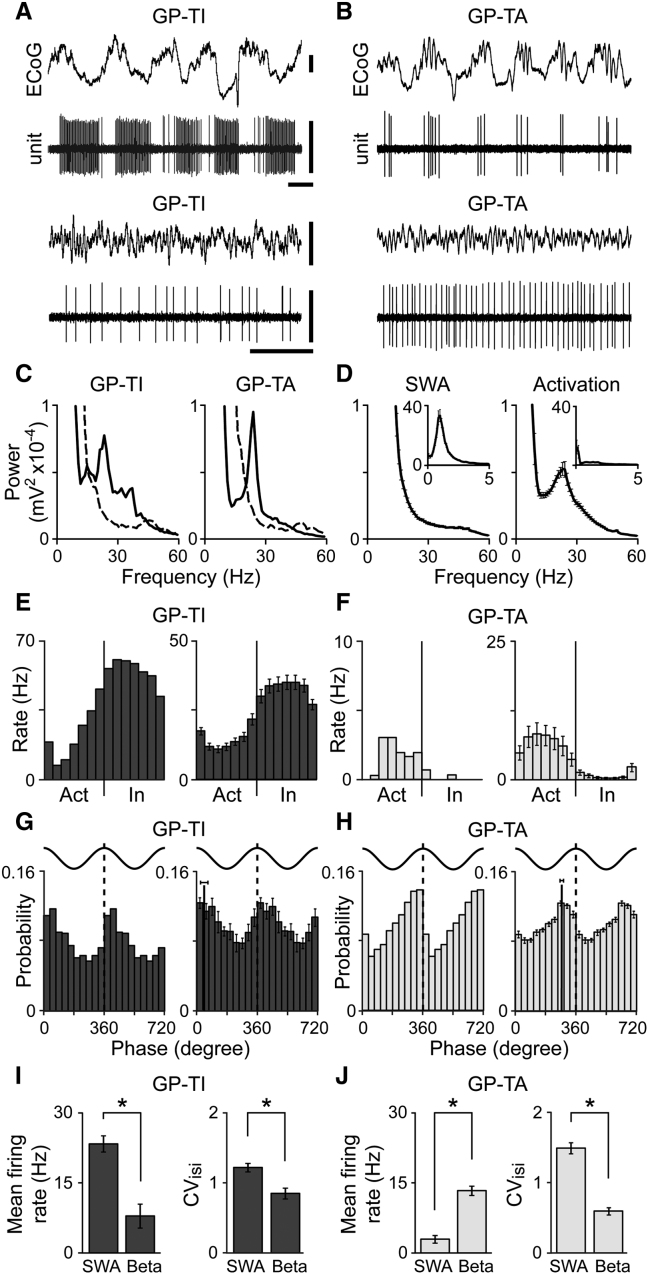
Dichotomous Firing Rates and Patterns of Identified Neurons in External Globus Pallidus (A and B) Typical single-unit activities of the two types of GPe neuron in 6-OHDA-lesioned Parkinsonian rats. The same GP-TI neuron (A) and GP-TA neuron (B) were recorded during cortical slow-wave activity (SWA, above) and activation (below), as defined in electrocorticograms (ECoG). Note their inversely-related firing patterns and rates. Single neuron data in (E)–(H) are from these same neurons. Vertical scale bars: 250μV (ECoG), 2mV (units). Horizontal scale bars: 0.5 sec. (C and D) Excessive beta oscillations (∼20 Hz) emerge in ECoGs recorded in the activated brain state. (C) ECoGs recorded with single neurons in (A) and (B) during SWA (dashed spectra) and activation (black spectra). (D) Mean spectra (±SEM) from ECoGs recorded with all GPe neurons. (E and F) Activity histograms of spike timings of GP-TI neurons (E) and GP-TA neurons (F) in relation to “active” (Act) and “inactive” (In) components of cortical slow oscillations. Mean histograms (±SEM) for all identified neurons of each type are on the right. (G and H) Phase histograms of spike timings of GP-TI neurons (G) and GP-TA neurons (H) with respect to peaks (0°/360°) of cortical beta oscillations, with two cycles shown for clarity. Mean histograms (±SEM) for all neurons of each type are on the right. Mean phase angles (±2 SEMs) are also indicated. (I and J) Mean firing rates and coefficients of variation of firing (CV_isi_) for all GP-TI neurons (I) and GP-TA neurons (J) during SWA and cortical beta oscillations. ^∗^p < 0.001 Mann-Whitney rank sum tests. Data are means ± SEM.

To define the functional roles played by physiologically distinct GPe neurons in vivo, we electrophysiologically characterized and juxtacellularly labeled (Magill et al., 2001; Pinault, 1996) single units (n = 79) in the GPe of 6-OHDA-lesioned, anesthetized adult rats (n = 45). We studied Parkinsonian rats because dopamine loss enhances physiological diversity in vivo (Magill et al., 2001; Mallet et al., 2006, 2008a). We analyzed the action potential discharges of these identified GPe neurons during two spontaneous brain states as determined from simultaneously-recorded frontal electrocorticograms: (1) slow-wave activity (SWA), which is similar to activity observed during natural sleep; and (2) “activation,” which contains activity patterns more analogous to those observed during the awake, behaving state (Mallet et al., 2008a, 2008b). In Parkinsonian rats, two major populations of GPe neurons are distinguished by their firing patterns during cortical SWA (Mallet et al., 2008a). When defined on the basis of physiological properties alone, most GPe neurons (∼75% of all spontaneously-active GPe units; Mallet et al., 2008a) preferentially discharge during the “inactive” (surface-negative) component of the cortical slow (∼1 Hz) oscillation, defined here as the part of the electrocorticogram cycle during which most cortical, striatal, and STN neurons are quiescent or least active (Mallet et al., 2006, 2008a, 2008b). These GPe units are thus called GP-TI neurons (Mallet et al., 2008a) (Figure 1A). In contrast, the second major population of GPe neurons (∼20% of all active GPe units; Mallet et al., 2008a) preferentially discharge during the “active” (surface-positive) component and are thus called GP-TA neurons (Mallet et al., 2008a) (Figure 1B). Using these diverse spike-timing relationships during SWA, we initially defined 86% of our recorded, labeled, and identified GPe neurons as either GP-TI neurons (n = 36) or GP-TA neurons (n = 32). The ratio of GP-TI and GP-TA neurons sampled with the relatively high-impedance glass electrodes used here does not match that which we previously reported for recordings made with low-impedance multielectrode arrays (Mallet et al., 2008a). The use of high-impedance electrodes, which were advanced with submicron precision, meant that we were better able to target GPe units with very low firing rates, thus shifting the ratio more in favor of GP-TA neurons (see below). Four identified GPe neurons did not fire in time with cortical slow oscillations but instead fired tonically at high firing rates (range: 6 to 27 Hz) (Mallet et al., 2008a); these were excluded from further analyses.

The two brain states studied here in Parkinsonian rats are defined by cortical oscillations of different frequencies and amplitudes. While SWA is dominated by large ∼1 Hz oscillations, the activated brain state instead favors the emergence of excessive oscillations at beta frequencies (15–30 Hz) (Mallet et al., 2008a, 2008b) (Figures 1A–1D). We examined whether the “antiphase” firing of GP-TI and GP-TA neurons was preserved across these two extreme brain states. For this purpose, some of the GP-TI and GP-TA neurons were also recorded during the activated brain state with its characteristic beta oscillations (Figures 1A and 1B). Dichotomous spike timings of GP-TI and GP-TA neurons during slow oscillations (Figures 1E and 1F) were indeed maintained during cortical beta oscillations (Figures 1G and 1H). GP-TI neurons (n = 14) were, on average, most likely to fire at 44.3° ± 18.4° (mean ± SEM; range of preferred angles 348°–134°, p < 0.05, Rayleigh tests) with respect to the peaks of cortical beta oscillations at 0°/360°. However, GP-TA neurons (n = 23) fired at a significantly different phase (p < 0.05, Watson-Williams *F* test) of 275.8° ± 7.4° (range, 208°–358° p < 0.05, Rayleigh tests). Average firing phases of these identified GP-TI and GP-TA neurons were similar to those of several hundred GPe units recorded with multielectrode arrays (Mallet et al., 2008a).

Different spike-firing patterns of identified neurons were mirrored by inversely-related firing rates, irrespective of brain state. During SWA, GP-TI neurons fired much faster than GP-TA neurons, but during beta oscillations, the situation was reversed and GP-TA neurons fired faster than GP-TI neurons (both p < 0.05, Mann-Whitney tests). Furthermore, most GP-TI neurons (93%) decreased their firing rates during transition from SWA to the activated brain state, whereas most GP-TA neurons (97%) increased firing (Figures 1I and 1J). Regularity of firing was also different, with GP-TI neurons firing more regularly than GP-TA neurons during SWA but less regularly during beta oscillations (both p < 0.05, Mann-Whitney tests). Both GP-TI and GP-TA neurons fired more regularly during beta oscillations as compared to SWA (Figures 1I and 1J).

Importantly, a small sample of identified GPe neurons (n = 7) fired so infrequently during SWA (mean firing rates of 0.002–0.2 Hz) that we could not statistically classify them as GP-TI or GP-TA neurons. However, we established that these very slow-firing neurons had some other key properties of GP-TA neurons. First, like GP-TA neurons, they all strongly increased their firing rate upon transition from SWA to the activated brain state (0.06 ± 0.02 Hz and 15.3 ± 2.4 Hz, respectively). Second, their firing was significantly modulated in time with cortical beta oscillations (p < 0.05, Rayleigh tests) and they were most likely to fire at phases (283.5° ± 10.5°; range, 246°–318°) that were similar to those preferred by identified GP-TA neurons but not by GP-TI neurons (p > 0.05 and p < 0.05, respectively, both Watson-Williams *F* tests). Thus, all of these slow-firing GPe cells were classified as GP-TA neurons for group analyses (the molecular profiles of these slow cells were also identical to GP-TA neurons; see below). These data suggest that the continuum of relatively low firing rates of GP-TA neurons during SWA extends to virtual quiescence. In summary, the great majority (up to 95%) of GPe neurons recorded in Parkinsonian rats can be assigned to one of two groups according to the rate, pattern, and mean phases of their firing during ongoing network oscillations.

### Physiological Dichotomy Is Mirrored by Molecular Dichotomy

**Figure 2:**
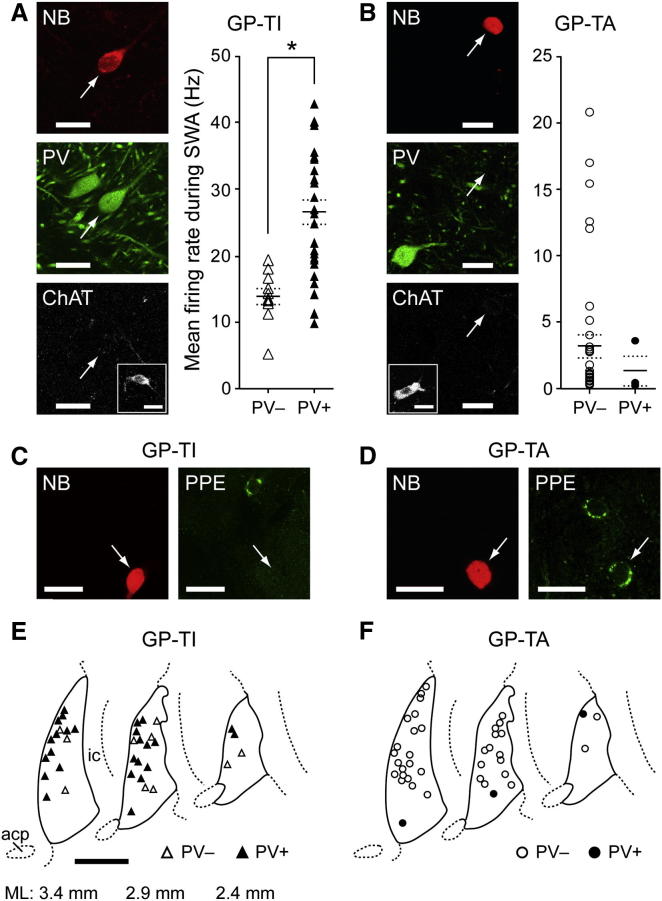
Electrophysiological Dichotomy of Identified Globus Pallidus Neurons Is Mirrored by Their Diverse Molecular Profiles After recording, single units were juxtacellularly labeled with neurobiotin (NB) and tested for expression of various molecular markers. (A) Confocal fluorescence micrographs of a typical GP-TI neuron that expressed parvalbumin (PV) but not choline acetyltransferase (ChAT). (B) Typical GP-TA neuron that did not express either PV or ChAT. Most GP-TI neurons expressed PV (PV+), whereas most GP-TA neurons did not (PV−). All identified GPe neurons tested in this study were ChAT− (insets show ChAT+ ventral pallidal neurons in the same focal plane acting as positive controls). (A and B) Firing rates of individual PV+ and PV− GPe neurons during slow-wave activity. Group means ± SEM are indicated by solid and dashed lines, respectively. ^∗^p < 0.001 Mann-Whitney rank sum test. (C) GP-TI neurons did not express preproenkephalin (PPE). (D) GP-TA neurons expressed PPE. (E and F) Schematics of parasagittal sections of GPe (delineated by solid lines) illustrating the approximate locations of the somata of all recorded and identified PV+ and PV− neurons in this study. ML (mediolateral) numbers denote positions with respect to midline. acp, anterior commissure; ic, internal capsule. Scale bars: (A–D) 20 μm; (E) 1.0 mm.

Full definition of a cell type requires correlation of temporal activity with neurochemistry and structure. Because we juxtacellularly labeled the GPe neurons with neurobiotin after their electrophysiological characterization, we could directly address the critical issue of whether physiological heterogeneity is reflected in molecular heterogeneity. The GPe is composed of GABAergic and cholinergic neurons. The small population of cholinergic neurons (∼5% of all GPe cells; Gritti et al., 2006) is usually not considered part of the BG per se, but rather an extension of the nucleus basalis of Meynert that is ventromedial and caudal of GPe. We tested large samples of identified GP-TI and GP-TA neurons (n = 17 and 30, respectively) for immunoreactivity for a cholinergic neuron marker, choline acetyltransferase (ChAT). None of the tested GPe neurons expressed ChAT, suggesting that GP-TI and GP-TA neurons are GABAergic (Kita, 2007; Smith et al., 1998) (Figures 2A and 2B). GABAergic GPe neurons are themselves molecularly diverse; most (∼60%) express the calcium-binding protein parvalbumin (PV), whereas the remainder express mRNA for a neuropeptide precursor, preproenkephalin (PPE) (Hoover and Marshall, 1999, 2002; Kita, 1994; Kita and Kita, 2001). Whether this molecular diversity correlates with different activity patterns in vivo (and whether GPe neurons actually make PPE protein) are unknown. We first tested all identified GPe neurons for PV immunoreactivity. Most GP-TI neurons (72%) expressed PV (PV+), whereas most GP-TA neurons (91%) did not (PV−) (Figures 2A and 2B). Moreover, PV+ GP-TI neurons fired faster during SWA than PV− GP-TI neurons (Figures 2A and 2B). Taken in context of the different population sizes as defined physiologically (75% GP-TI versus 20% GP-TA units; Mallet et al., 2008a), this result indicates that >95% of PV+ GPe neurons are GP-TI neurons, whereas an individual PV− neuron will have an approximately equal chance of being either a GP-TI or GP-TA neuron. Thus, PV is a selective (not specific) marker of the in vivo physiological phenotype of GABAergic GPe neurons. We next tested for the expression of PPE protein in both populations of GPe neuron. None of the tested GP-TI neurons (n = 19) expressed PPE (Figure 2C), regardless of PV expression (n = 15 PV+ and 4 PV− neurons). In contrast, all tested GP-TA neurons (n = 9; all PV−) expressed PPE protein, evident as punctate cytoplasmic immunoreactivity (Figure 2D). This suggests that, within GPe cells, PPE is a specific molecular marker for GP-TA neurons. Our juxtacellular labeling also showed that GP-TI and GP-TA neurons are intermingled and distributed throughout GPe (Figures 2E and 2F). Together, these electrophysiological and neurochemical data show that GPe diversity correlates across functional levels, with PPE−(PV+) GABAergic neurons (GP-TI) exhibiting inversely-related firing patterns/rates with PPE+(PV−) GABAergic neurons (GP-TA).

### Structural Dichotomy Reveals the Existence of a Novel Cell Type in External Globus Pallidus

**Figure 3:**
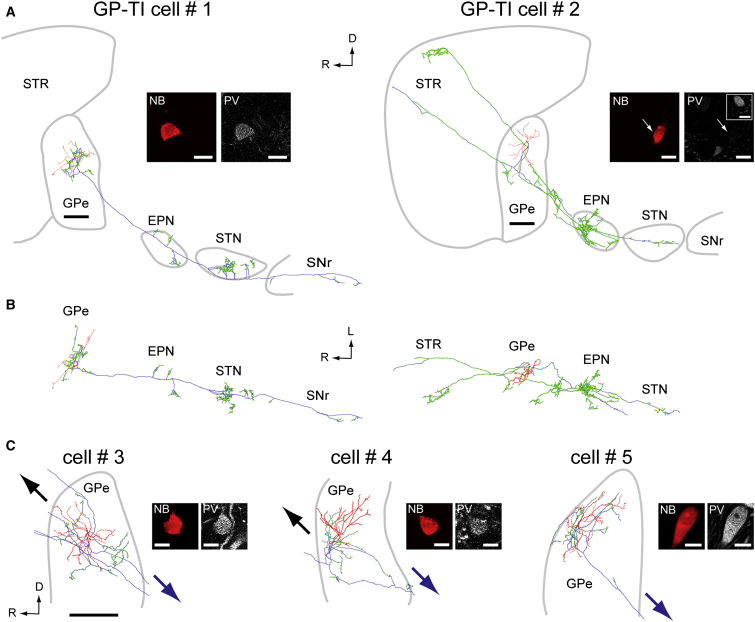
GP-TI Neurons Have the Prototypic Structure of Globus Pallidus Neurons (A and B) Full reconstructions of two neurobiotin-labeled GP-TI neurons in parasagittal (A, medial view) and horizontal planes (B, dorsal view). Somata and dendrites are drawn in red, axons in blue, and axonal boutons in green. Each neuron was prototypic in its long-range axonal projections descending to the subthalamic nucleus (STN) and other basal ganglia nuclei (EPN, entopeduncular nucleus; SNr, substantia nigra pars reticulata). Each neuron also gave rise to extensive local axon collaterals in external globus pallidus (GPe), and some cells additionally innervated striatum (STR). Confocal fluorescence micrographs illustrate tests for co-localization of the neurobiotin (NB) signal with immunoreactivity for parvalbumin (PV). Inset in (A) shows another PV+ GPe neuron in the same focal plane as Cell #2 acting as a positive control. (C) Three more identified GP-TI neurons, with only somata, dendrites, local axon collaterals, and proximal extrinsic projections reconstructed. Arrows indicate directions of long-range axonal projections as they exited GPe (“upstream” in black, “downstream” in blue). Scale bars: 0.5 mm for all reconstructions, and 20 μm for all fluorescence micrographs. R, rostral; D, dorsal; L, lateral.

**Figure 4:**
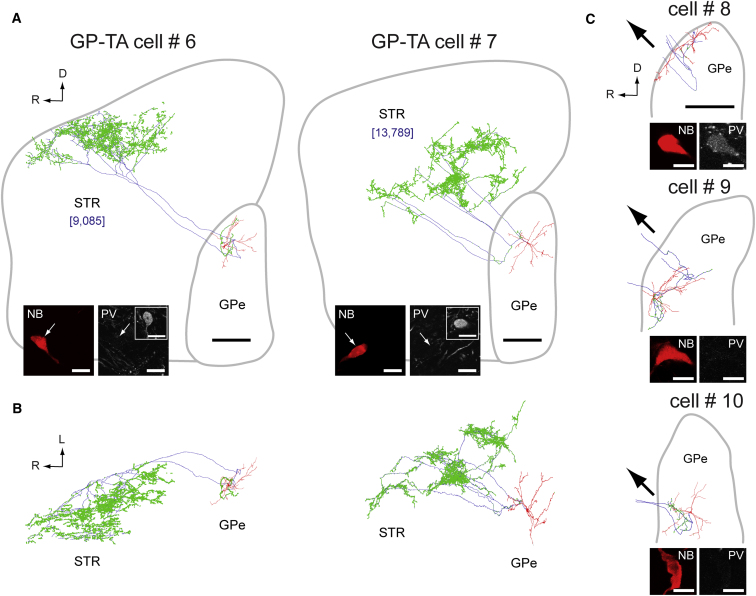
GP-TA Neurons Have Unique Structural Properties and Are a Novel Cell Type in the Globus Pallidus (A and B) Full reconstructions of two neurobiotin-labeled GP-TA neurons in parasagittal (A, medial view) and horizontal planes (B, dorsal view). Somata and dendrites are drawn in red, axons in blue, and axonal boutons in green. The long-range axonal projections of each neuron provided a massive, dense, and specific innervation of striatum (STR). Numbers of axonal boutons in striatum are given in blue brackets. Confocal fluorescence micrographs illustrate tests for co-localization of the neurobiotin (NB) signal with immunoreactivity for parvalbumin (PV). Insets in (A) show other PV+ GPe neurons in the same focal planes acting as positive controls. (C) Three more identified GP-TA neurons, with only somata, dendrites, local axon collaterals, and proximal extrinsic projections reconstructed. Black arrows indicate direction of long-range axonal projections as they exited GPe (only “upstream” to striatum). Scale bars: 0.5 mm for all reconstructions, and 20 μm for all fluorescence micrographs. R, rostral; D, dorsal; L, lateral.

Functional duality in GPe has major implications for the expression of both pathological and normal activities in BG circuits. To better understand potential cell-type-specific contributions to the propagation of excessive beta oscillations and other activities to BG nuclei, we next defined the axonal and dendritic architecture of identified GP-TI neurons and GP-TA neurons. To achieve this, neurobiotin-labeled processes of individual neurons were visualized with a permanent reaction product formed by nickel-diaminobenzidine (Ni-DAB) and then digitally reconstructed (persons executing reconstructions were blind to electrophysiological phenotype). We first focused on the long-range and local axonal projections of some well-labeled cells. We thus reconstructed in three dimensions the entire axonal arborizations of two GP-TI neurons (cells #1 and #2, Figures 3A and 3B) and of two GP-TA neurons (cells #6 and #7, Figures 4A and 4B). We also reconstructed the local axon collaterals and proximal extrinsic projections of three more GP-TI neurons (cells #3, #4, and #5, Figure 3C) and three more GP-TA neurons (cells #8, #9, and #10, Figure 4C). During the digital reconstruction process, we marked all axonal boutons. Because >96% of these large pallidal boutons form at least one synapse (Baufreton et al., 2009; Sadek et al., 2007), we used bouton counts to accurately estimate the degree of synaptic innervation of each target nucleus by each reconstructed GPe neuron. Importantly, all reconstructed GP-TI neurons (five cells, four of which were PV+) gave rise to extensive local axon collaterals and at least one long-range projecting axon collateral that descended beyond caudoventral GPe boundaries (Figure 3). The major targets of this descending projection were multiple “downstream” BG nuclei, including the EPN, STN, and SNr (Figures 3A and 3B). The fully-reconstructed GP-TI cells #1 and #2 gave rise to, respectively, 131 and 1311 boutons in EPN, 159 and 149 boutons in STN, and (for cell #1 only) 32 boutons in SNr. With respect to extrinsic projections then, GP-TI neurons thus have the definitive connections of prototypic GPe neurons (Smith et al., 1998). However, as well as emitting a descending projection axon, some GP-TI neurons also emitted ascending collaterals that modestly innervated striatum (Figures 3A and 3C) (Bevan et al., 1998; Kita and Kita, 2001; Kita and Kitai, 1994). The ascending axon of GP-TI cell #2 formed 621 boutons in striatum. This bouton count and those in STN are well within the ranges reported for single GPe neurons in dopamine-intact animals (Baufreton et al., 2009; Bevan et al., 1998).

Our reconstructions revealed that GP-TA neurons have a surprising and unique structure (Figure 4). Unlike prototypic GP-TI cells, none of the GP-TA neurons (n = 5) gave rise to a descending projection axon that targeted downstream BG nuclei. Instead, all reconstructed GP-TA neurons emitted local axon collaterals (although somewhat restricted; see below) and at least one ascending projection axon collateral that targeted striatum (Figure 4). Reconstructing the full axonal arborizations of every labeled GP-TA neuron was beyond the scope of this study, but we visually confirmed the extrinsic axonal projections of another nine GP-TA neurons (revealed with Ni-DAB). All but one of these neurons gave rise to only ascending axonal projections that entered and ramified in striatum; one unusual neuron innervated striatum and EPN. GP-TA neurons thus challenge the widely-held assumption that all GPe neurons innervate STN (Baufreton et al., 2009; Bevan et al., 1998; Smith et al., 1998; Wichmann and DeLong, 1996). The specific striatal innervation of the two fully-reconstructed GP-TA neurons (cells #6 and #7) was massive; the main axon split to form up to five ascending collaterals that established dense clusters of boutons over large striatal territories (Figures 4A and 4B). Remarkably, each GP-TA neuron gave rise to thousands of axonal boutons in striatum (9,085 and 13,789 boutons for cells #6 and #7). This extensive striatal innervation meant that total axon lengths of GP-TA neurons (126.2 and 164.2 mm for cells #6 and #7) were considerably longer than those of GP-TI neurons (37.8 and 59.4 mm for cells #1 and #2). Electrophysiological and molecular diversity in GPe is thus mirrored by a profound structural diversity. While GP-TI neurons innervate STN and other downstream BG nuclei, GP-TA neurons do not conform to this prototypic connectivity but instead provide a massive innervation of striatum.

### Cellular Targets of GP-TA Neurons that Exclusively Innervate Striatum

**Figure 5:**
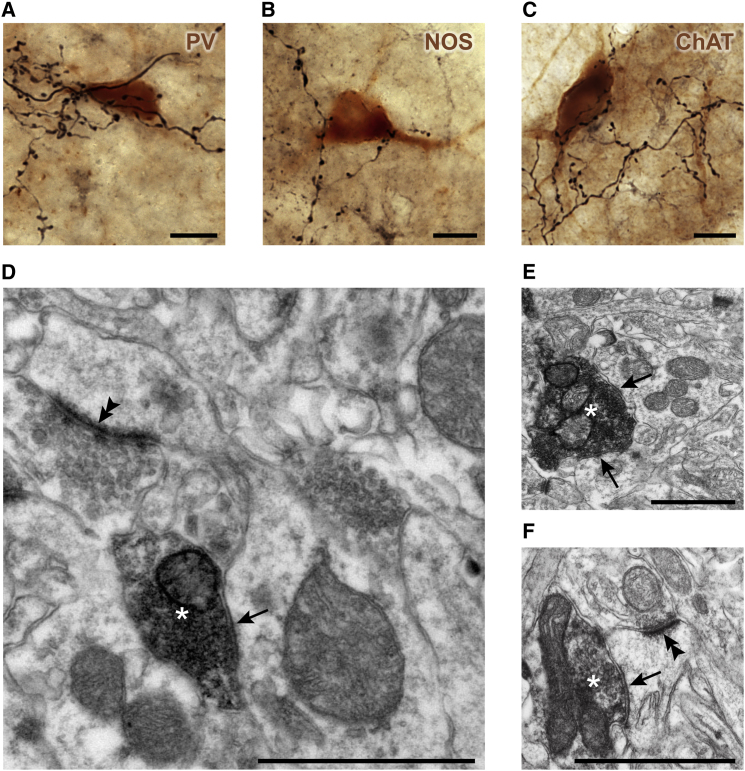
GP-TA Neurons Target Interneurons and Projection Neurons in Striatum (A–C) Extended-focus light micrographs of GP-TA neuron axons, revealed by a black precipitate, in close apposition to the somata and proximal dendrites of striatal interneurons expressing parvalbumin (PV), nitric oxide synthase (NOS), or choline acetyltransferase (ChAT), as revealed by brown precipitate. (D–F) Electron micrographs of GP-TA neuron axon terminals (asterisks) making symmetrical synaptic contacts (arrows) with the dendritic shafts of spiny striatal neurons (D and E) or the neck of a dendritic spine (F). The heads of spines in (D and F) formed asymmetrical synapses (double arrowheads) with unidentified axon terminals. Note that spines emanating from the dendrite in (E) were identified in serial sections. Scale bars: (A–C) 10 μm; (D–F) 1 μm.

Our discovery of a novel GPe cell type that only projects to striatum raises the issue of which types of striatal neuron are innervated. We first tested whether identified GP-TA neurons target the three major classes of aspiny interneuron (two GABAergic, one cholinergic) by revealing immunoreactivity for PV, nitric oxide synthase (NOS), or ChAT (Tepper and Bolam, 2004), respectively, with a light-brown DAB precipitate. The axons of single neurobiotin-labeled GP-TA neurons (n = 3) were revealed with a black Ni-DAB precipitate. Axonal boutons were found in close apposition to the somata and proximal dendrites of all three classes of interneuron, some of which were targeted by clusters of apposed boutons arranged in a “basket-like” manner (Figures 5A–C). Such specialized arrangements are indicative of synaptic contacts established by GPe cells (Bevan et al., 1998; Sadek et al., 2007). This analysis thus suggests that different classes of striatal interneurons are targeted by GP-TA neurons. However, we observed that many boutons were not apposed to these interneurons, so we next tested whether the axons of GP-TA neurons could establish synaptic contacts with striatal projection neurons (MSNs). Our electron microscopic analysis of the axon terminals of GP-TA neurons (38 boutons selected at random from three cells) revealed ultrastructural features typical of those of other GPe neurons (Smith et al., 1998), i.e., relatively large (>1 μm) and usually containing several mitochondria (Figures 5D–F). These axon terminals established short, symmetrical (Gray's Type II) synaptic contacts with the shafts of spine-bearing dendrites (32% of synapses; Figures 5D and 5E) and the necks/heads of spines (21%; Figure 5F), therefore indicating that MSNs are targeted. Other targets included dendritic shafts of striatal neurons that could not be unequivocally identified as MSNs in serial ultrathin sections (42%; see Experimental Procedures). Thus, GP-TA neurons are a novel source of GABA in striatum that is directed to projection neurons and major interneuron populations.

### Local Axonal Connectivity and Dendritic Architecture

**Figure 6:**
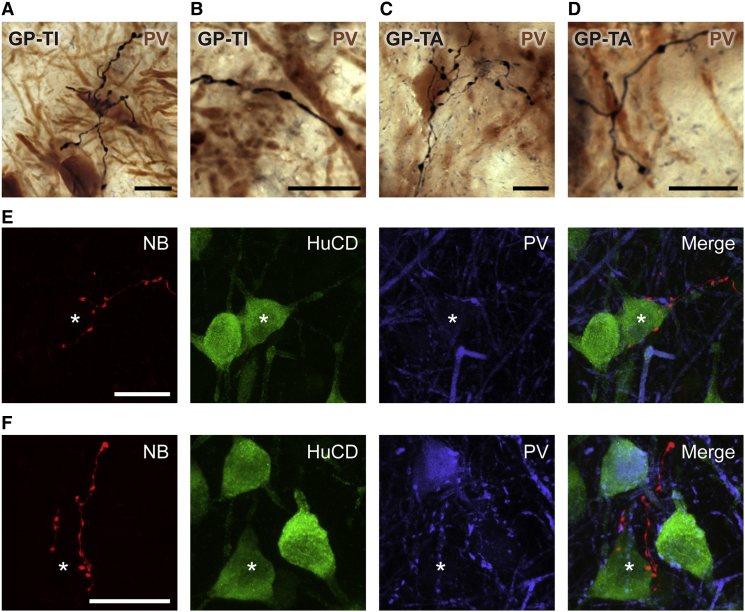
Local Axons of Different Types of Globus Pallidus Neuron Support Multiple Modes of Reciprocal GABAergic Influence (A and B) Extended-focus light micrographs of identified GP-TI neuron axon collaterals (black) in close apposition to somata (A) and dendrites (B) of neighboring parvalbumin (PV)-expressing GPe neurons (brown), i.e., putative GP-TI neurons. (C and D) Identified GP-TA neuron axon collaterals (black) closely apposed to a soma (C) and dendrites (D) of neighboring PV+ GPe neurons (brown). (E and F) Confocal fluorescence micrographs of neurobiotin-labeled axon collaterals (NB) of GP-TI neurons targeting neighboring GPe neurons that express the pan-neuronal marker HuCD but not PV (asterisks), i.e., putative GP-TA neurons. Scale bars: (A–D) 10 μm; (E and F) 20 μm.

To test whether GP-TI and GP-TA neurons could establish mutual connections, we next analyzed the local axon collaterals of identified single neurons. On average, GP-TI neurons gave rise to significantly longer local axon collaterals, with a larger number of boutons, than GP-TA neurons (Table 1). The bouton counts on local axon collaterals of GP-TI neurons are well within the ranges reported for single GPe neurons labeled in dopamine-intact animals (Sadek et al., 2007). To test for connections between GPe neurons of the same type and for connections between different types of neuron, we took advantage of the differential expression of PV by GP-TI and GP-TA neurons. We first addressed whether GP-TI and GP-TA neurons could contact PV+ (putative GP-TI) neurons. Some of the local axonal boutons of GP-TI neurons (1078 boutons selected at random from three cells) were closely apposed to the somata (5.2 ± 3.0%) or proximal dendrites (12.2 ± 7.0%) of PV+ GPe neurons (Figures 6A and 6B). A similar scenario held for GP-TA neurons (440 boutons analyzed from five cells), with some boutons targeting somata (4.8 ± 2.0%) or proximal dendrites (16.9 ± 6.0%) of PV+ GPe neurons (Figures 6C and 6D). Finally, we qualitatively determined whether GP-TI neurons could target GP-TA neurons. We did not use PPE immunoreactivity to unequivocally identify GP-TA neurons because our “antigen retrieval” protocol (see Experimental Procedures) compromised neurobiotin labeling in fine axon collaterals. Instead, we used triple fluorescence labeling to visualize all GPe neurons (expressing the pan-neuronal marker HuCD), those that were PV+, and the axons of single GP-TI neurons. Local axon collaterals of GP-TI neurons could indeed closely appose perisomatic regions of HuCD+/PV− (putative GP-TA) neurons (Figures 6E and 6F). These data demonstrate that GP-TI and GP-TA neurons make distinct contributions to a complex network of local connections, and reveal several modes of reciprocal GABAergic influence in GPe.

The GP-TI and GP-TA neurons that we studied in detail (cells #1–10) gave rise to two to seven primary dendrites that branched repeatedly and extended over large distances (total dendritic lengths of 7.66 ± 0.37 mm and 7.36 ± 0.71 mm, respectively). The two cell types were indistinguishable in terms of number of primary dendrites and dendritic segments, total dendritic length, and highest branch order (data not shown). All neurons bore dendritic spines (of various forms), and highly-varicose branching ramifications (Bevan et al., 1998; Sadek et al., 2007) were present at some distal dendrites. On average, the dendrites of GP-TA neurons bore spines at a significantly higher density than those of GP-TI neurons (Table 1). Altogether, our anatomical analyses of GP-TI and GP-TA neurons showed that physiological dichotomy in GPe is supported by cell-type-specific differences in the structure of dendrites, local axon collaterals, and, most strikingly, long-range axonal projections.

## Discussion

We provide the first direct correlation of the electrophysiological properties of individual GPe neurons in vivo with their molecular profiles and structure. In doing so, we elucidate key features that together constitute the foundations of a dichotomous functional organization of GPe. Two GPe cell types are thus specialized to release GABA, with or without a neuropeptide, on largely distinct BG neuronal populations in different temporal patterns according to brain state.

Neurons of the same cell type deliver identical neuroactive substances to a matching range of postsynaptic targets in the same temporal patterns (Somogyi, 2010). Our data are unique in establishing that GP-TI and GP-TA neurons are different cell types as defined at several requisite levels of function. Our electrophysiological recordings readily distinguished two GABAergic GPe neuron populations with distinct neurochemical and structural properties. Most GP-TI neurons express PV, whereas almost all GP-TA neurons do not. While GP-TA neurons express PPE protein, suggesting they use enkephalin as a co-transmitter, GP-TI neurons do not. This physiological and molecular diversity is mirrored in cell structure. Thus, GP-TI neurons are prototypic in always innervating downstream BG nuclei like STN, whereas GP-TA neurons exclusively provide a massive input to striatum.

The diverse electrophysiological properties of GPe neurons (Kita, 2007; Mallet et al., 2008a) suggest different functions, but to firmly establish this, physiological diversity must be put into context with structure. By correlating spike timing in vivo with neurochemistry and outputs, we provide a good working definition of a functional dichotomy in GPe. Examination of synaptic transmission dynamics, causal interactions, and other parameters in the future will help to fully characterize this dichotomy. Molecular and structural diversity of GPe neurons has been reported at the population level (Kita, 2007) but has not been related to activity in vivo. Moreover, anatomical studies carried out at the population level neither fully define a cell type nor demonstrate the novel organizational features that we detail here for individual neurons. That said, our results help tally findings of population-level studies: >97% of PV+ GPe neurons do not express PPE mRNA and vice versa (Hoover and Marshall, 1999); PV+ GPe cells often project to downstream targets but PPE mRNA^+^ cells often do not (Hoover and Marshall, 1999); and many PV− GPe neurons project to striatum (Kita and Kita, 2001). Preproenkephalin mRNA^+^ cells make up ∼40% of all GPe neurons (Hoover and Marshall, 2002; Voorn et al., 1999), yet large-scale extracellular recordings with low-impedance multielectrode arrays show that GP-TA neurons constitute ∼20% of active GPe units (Mallet et al., 2008a). The latter approach might under-sample this cell population, and, indeed, we identified virtually quiescent GP-TA neurons that would be difficult to detect using this technique. When sampling biases are considered, it might be the case that GP-TA neurons are almost as numerous as prototypic GP-TI neurons. GP-TA and GP-TI neurons are located throughout GPe, suggesting that they lie in the sensorimotor, associative, and limbic domains of this nucleus (Smith et al., 1998).

Single-cell labeling allowed us to reveal the existence of a novel GPe cell type with unique properties that cannot be determined from population-level studies. The structure of these GP-TA neurons is remarkable because they do not innervate STN but instead provide a massive input to striatum. Their lack of descending projection axons is not the result of incomplete labeling with neurobiotin. The axons of GP-TA neurons were well labeled, their lengths far exceeding those of GP-TI neurons. As such, the properties of GP-TA neurons challenge the idea that an essential function of all GPe neurons is to inhibit STN neurons (Albin et al., 1989; Bevan et al., 2002; Smith et al., 1998; Wichmann and DeLong, 1996). From our sample of fully-reconstructed neurons, we estimate that each GP-TA neuron gives rise to ∼10,000 axonal boutons directed to wide expanses of striatum. Individual GP-TA neurons thus provide the largest GABAergic innervation of striatum of any known (quantified) cell type. As a population, GP-TA neurons also represent the largest extrinsic source of GABA in striatum. Indeed, their striatal boutons are > 10 times more abundant than those of GPe cells that also target downstream BG (Bevan et al., 1998). The pallidostriatal axons of the latter, which are likely GP-TI neurons, selectively target GABAergic interneurons (Bevan et al., 1998). Whether this type of GPe neuron also innervates striatal projection neurons is unknown. Importantly, however, we show here for the first time that some identified GPe neurons (i.e., the GP-TA neurons) can form synaptic contacts with striatal MSNs. We also establish that GP-TA neurons can additionally target two types of striatal GABAergic interneurons (PV+, NOS+) as well as cholinergic interneurons. GABA release from GP-TA neurons is thus well suited to control activity in striatal circuits. Moreover, GP-TA neurons are potentially a second important source of enkephalin in striatum, the first being PPE+ MSNs of the indirect pathway (Blomeley and Bracci, 2011; Gerfen and Surmeier, 2011). Enkephalin released from the dense terminal fields of GP-TA neurons could act at mu opioid receptors on corticostriatal afferents to reduce glutamatergic drive of MSNs (Blomeley and Bracci, 2011). Opioidergic effects of GP-TA cells would thus complement a direct GABAergic inhibition of MSNs, with potential selectivity for striatal striosomes/patches versus matrix (Crittenden and Graybiel, 2011). Because GP-TA neurons can cast broad nets of influence over striatum, we call them “arkypallidal” neurons (from ancient Greek ἄρκυς [arkys] for “hunter's net”). Understanding precisely how arkypallidal neurons fit into the direct/indirect pathways model or any other scheme of BG organization is a key challenge. Although beyond the scope of this study, it would be important in the future to determine whether arkypallidal neurons selectively innervate MSNs of the indirect pathway or the direct pathway. Selective innervation of the former (striatopallidal) neurons could provide a substrate for closed-loop feedback that would have to be carefully controlled in order to avoid excessive activity of either GABAergic partner. On the other hand, selective targeting of MSNs that innervate BG output nuclei could mediate a novel mode of open-loop inhibition in striatum; arkypallidal neurons could thus dampen the activity of direct pathway MSNs until they themselves were inhibited by striatopallidal neurons. Widespread but non-selective innervation of both types of MSN by arkypallidal neurons could alternatively subserve an activity pattern akin to an “all stop” signal to striatum. Of course, the balance of activity in these circuits would also critically depend on whether arkypallidal neurons preferentially target striatal projection neurons rather than interneurons. In short, our data suggest that any controlling input to arkypallidal neurons is, by virtue of the unique properties of this cell type, well positioned to powerfully influence one or the other or both of the output pathways of striatum.

In contrast to arkypallidal neurons, GP-TI neurons infrequently innervate striatum but always target downstream BG nuclei like STN. Individual GPe neurons (of unknown neurochemistry) with descending and ascending projection axons have been described in dopamine-intact animals (Bevan et al., 1998; Kita and Kitai, 1994), emphasizing the widespread influence that a single GPe (GP-TI) neuron can have on the BG. Our reconstructed GP-TI neurons show that, innervation of STN aside, there is considerable variety in the selectivity and size of their innervation of other BG nuclei. Taken in light of previous findings in rodents and monkeys (Bevan et al., 1998; Kita and Kitai, 1994; Sato et al., 2000), prototypic GPe neurons can thus additionally target EPN and SNr, or EPN but not SNr or vice versa. No model of BG organization adequately captures this rich structural diversity in the outputs of individual neurons or networks of GPe. Nevertheless, the distinct properties of prototypic and arkypallidal neurons imply that they fulfill specialized, broadly complementary roles in BG circuits, such as gating cortical inputs to STN or striatum, respectively.

During both SWA and activated brain states, prototypic and arkypallidal neurons are distinguished by inversely-related firing rates and patterns, as well as by their preferred phases of firing during slow (∼1 Hz) and beta (15–30 Hz) oscillations. Prototypic GP-TI neurons fire with appreciable phase differences (“antiphase”) compared to STN and striatal neurons (Magill et al., 2001; Mallet et al., 2006, 2008a, 2008b), while arkypallidal neurons fire in-phase with these major afferents. Synchronized neuronal oscillations play key roles in normal brain function (Buzsáki and Draguhn, 2004; Singer, 1999), with abnormal or uncontrolled synchronization accompanying many cognitive and motor disorders (Schnitzler and Gross, 2005; Uhlhaas and Singer, 2006). This is exemplified in Parkinsonism, in which “antikinetic” excessive beta oscillations emerge in every BG nucleus (Avila et al., 2010; Brown et al., 2001; Hammond et al., 2007; Mallet et al., 2008a, 2008b; Moran et al., 2011). Our analyses provide critical new insights into how GPe neurons might coordinate and propagate beta oscillations across basal ganglia circuits in a cell-type-specific manner. First, antiphase rhythmic activities of reciprocally-connected GABAergic GP-TI and glutamatergic STN neurons could effectively reinforce beta oscillations. Second, although arkypallidal and STN neurons synchronize at beta frequencies, the former cannot directly influence the latter, as suggested by recent computational modeling (Cruz et al., 2011). However, arkypallidal neurons could directly influence the rhythmic activity of GP-TI neurons (and vice versa) through local axon collaterals and, indeed, these cell types are synchronized at beta frequencies (Cruz et al., 2011; Mallet et al., 2008a). The precise operations mediated by the reciprocal connections of prototypic and arkypallidal neurons are unclear, but, in theory, these local GABAergic inputs could reduce target activity by membrane hyperpolarization, provide “shunting” inhibition, drive activity through rebound responses, and/or phase-lock and synchronize target activity. With the latter in mind, it is tempting to hypothesize that the complex local connections of GPe neurons enable the Parkinsonian network to act as a central pattern generator for beta oscillations. Third, GP-TI neurons are a single-cell substrate for entraining neuronal activity in every BG nucleus. Fourth, arkypallidal neurons are exceptionally suited to impose beta oscillations on large, spatially-distributed ensembles of diverse striatal neurons. Corollary predictions are that prototypic and arkypallidal neurons collectively exert profound influence over the BG, for better or worse, and that therapeutic interventions targeted to GPe should ameliorate PD symptoms. Indeed, “deep brain stimulation” delivered in GPe of PD patients has clinical benefit (Vitek et al., 2004), which cannot be readily explained by classic models of BG organization (Albin et al., 1989; Smith et al., 1998; Wichmann and DeLong, 1996).

Neurochemical and structural changes to BG neurons, including axon reorganization, abound in PD and its animal models, usually as homeostatic responses to redress pathological changes (Gerfen and Surmeier, 2011; Gittis et al., 2011). Possible axon retraction in arkypallidal neurons would not support homeostasis because it would exacerbate pathological STN hyperactivity in Parkinsonism (Mallet et al., 2008a, 2008b). Besides, axons of GP-TI neurons were quantitatively similar to those of individual GPe neurons in dopamine-intact rats (Baufreton et al., 2009; Bevan et al., 1998; Sadek et al., 2007), further arguing against macroscale reorganization of GPe axons in this PD model. Additional evidence suggests that the molecular and structural duality characterized here extends beyond Parkinsonism and beyond rodents. Indeed, PV−/PPE mRNA^+^ GPe neurons that preferentially project to striatum are abundant in dopamine-intact animals (Hoover and Marshall, 1999; Voorn et al., 1999). Moreover, single-axon tracing in primates suggest that 15%–20% of GPe axons collateralize in striatum only, with the remainder targeting at least STN (Sato et al., 2000), although this approach is somewhat ambiguous. Physiologically-defined GP-TI and GP-TA neurons also exist in dopamine-intact rats, but in much smaller proportions (Mallet et al., 2008a). One might speculate that the physiological diversity in rodent GPe is related to that recorded in GPe in behaving monkeys; prototypic and arkypallidal neurons might correspond to the primate GPe units that exhibit “high-frequency discharge” (but with pauses) or “low-frequency discharge and bursts,” respectively (DeLong, 1971, 1972; Elias et al., 2007). So why does physiological dichotomy come to the fore in PD? Markers of activity in PV+ and PV− (PPE mRNA^+^) GPe neurons are positively and negatively modulated, respectively, by dopaminergic transmission (Billings and Marshall, 2004; Hoover and Marshall, 2002). Chronic dopamine loss could therefore imbalance the activities of these diverse GPe neurons, teasing them further apart, much as it does in striatum in PD (Gerfen and Surmeier, 2011).

Striatal projection neurons exhibit prominent functional duality, a fundamental circuit feature conserved from the phylogenetically-oldest group of vertebrates (Stephenson-Jones et al., 2011). Thus, direct and indirect pathway MSNs have distinct electrophysiological properties, neurochemistry and axonal projections, as well as opposing influences on behavior (Gerfen and Surmeier, 2011). We provide evidence for a dichotomous functional organization of GPe that is as compelling as that in striatum. Because all vertebrate brains likely have (homologs of) both striatum and pallidum (Stephenson-Jones et al., 2011), one intriguing possibility is that functional duality co-evolved across the striato-pallidal axis. Given our new findings, GPe circuits are realistically viewed not as a single, homogeneous entity but as two interacting systems that engage in a division of labor to orchestrate both normal and abnormal activities across the entire basal ganglia.

## Experimental Procedures

Experimental procedures were carried out on adult male Sprague-Dawley rats (Charles River), and were conducted in accordance with the United Kingdom Animals (Scientific Procedures) Act, 1986. See Supplemental Experimental Procedures for further details.

### 6-Hydroxydopamine Lesions of Dopamine Neurons

Unilateral 6-hydroxydopamine (6-OHDA) lesions were induced in 190–305 g rats, as described previously (Mallet et al., 2008a, 2008b). All animals received desipramine (25 mg/kg, i.p.; Sigma) to minimize the uptake of 6-OHDA by noradrenergic neurons (Schwarting and Huston, 1996b). Lesions were assessed after 6-OHDA injection by challenge with apomorphine (0.05 mg/kg, s.c.; Sigma) (Schwarting and Huston, 1996a). Electrophysiological recordings were carried out in the GPe ipsilateral to 6-OHDA lesions in anesthetized rats 21–45 days after surgery.

### In Vivo Electrophysiological Recording and Juxtacellular Labeling of Single Neurons

Recording and labeling experiments were performed in 45 anesthetized 6-OHDA-lesioned rats (271–540 g at the time of recording). Anesthesia was maintained with urethane (1.3 g/kg, i.p.), and supplemental doses of ketamine (30 mg/kg, i.p.) and xylazine (3 mg/kg, i.p.), as described previously (Mallet et al., 2008a, 2008b). The epidural ECoG was recorded above the frontal (somatic sensory-motor) cortex (Mallet et al., 2008a). Extracellular recordings of single-unit activity in the GPe were made using glass electrodes (11–29 MΩ in situ; tip diameter ∼1.2 μm) containing 0.5 M NaCl solution and neurobiotin (1.5% w/v; Vector Laboratories). Following electrophysiological recordings, single neurons were juxtacellularly labeled with neurobiotin (Magill et al., 2001; Mallet et al., 2008a; Pinault, 1996). Seventy-nine individual GPe neurons were juxtacellularly labeled in this study.

### Tissue Processing for Light Microscopy

Parasagittal sections (50 μm) were cut from each perfusion-fixed brain and incubated overnight in Cy3-conjugated streptavidin. Sections containing neurobiotin-labeled neuronal somata (those marked with Cy3) were then isolated for molecular characterization by indirect immunofluorescence. All identified GPe neurons were tested for expression of parvalbumin (PV), and some were also tested for choline acetyltransferase (ChAT) and/or preproenkephalin (PPE). To optimize immunolabeling for PPE, that is, to readily distinguish PPE+ GPe neurons from the high levels of PPE immunoreactivity in striatopallidal axons, we used a heat pre-treatment as a means of antigen retrieval (Jiao et al., 1999). In some experiments, we also revealed neighboring GPe neurons by immunoreactivity for human neuronal protein HuC/HuD (HuCD). Neurochemical verification was performed by assessing immunofluorescence in single-plane confocal images. A neuron was classified as not expressing the tested molecular marker only when positive immunoreactivity could be observed in other cells on the same focal plane as the tested neuron. To visualize the somatodendritic and axonal architecture of identified neurons using brightfield microscopy, we then revealed the neurobiotin tracer with a permanent reaction product, Ni-DAB (Magill et al., 2001; Sadek et al., 2007). When targets of GPe neurons were to be identified, sections not containing Ni-DAB-labeled somata were further processed by the “peroxidase-anti-peroxidase” method to reveal other neurons expressing PV, nitric oxide synthase, or ChAT with a DAB reaction product (Bevan et al., 1998).

### Digital Reconstruction of the Dendritic and Axonal Architecture of Single Neurons

Reconstructions were performed blind to electrophysiological phenotype. Five identified GP-TI neurons and five GP-TA neurons (cells #1–10; see Figures 3 and 4) were traced in three dimensions using Neurolucida software (MBF Bioscience) (Sadek et al., 2007). Morphometric analyses were carried out using Neurolucida Explorer software (MBF Bioscience).

### Tissue Processing for Electron Microscopy

Electron microscopy was carried out according to standard protocols (Sadek et al., 2007), and was only performed if just one GP-TA neuron was juxtacellularly labeled in the brain. After examination in the light microscope, pieces of striatal tissue containing the axonal arborizations of GP-TA neurons were dissected out. Serial ultrathin sections (∼50 nm) were cut and, for labeled axon terminals forming synapses, images of serial sections (up to 10) were recorded. The striatal structures postsynaptic to GPe axon terminals (i.e., dendritic shafts or spines) were characterized. Spines were identified on the basis of their emergence from a dendritic shaft, their relatively small size, the absence of mitochondria, and/or the presence of spine apparatus.

### Electrophysiological Data Analysis

The classification of GPe units recorded during slow-wave activity as either “GP-TI” or “GP-TA” was performed by computing the “activity histogram” of single-unit activity with respect to the cortical slow (∼1 Hz) oscillation (Mallet et al., 2008a). The coefficient of variation of the interspike interval (CV_isi_) was calculated as an indicator of firing regularity. Linear phase histograms were used to examine the temporal relationships between the firing of identified GPe neurons and cortical beta oscillations (Mallet et al., 2008a). Modulations of unit activity in time with cortical beta oscillations were tested for significance using Rayleigh's Uniformity Test (Oriana; Kovach Computing). The mean angle of spike firing, with respect to the peaks of the cortical oscillation (defined as 0°/360°), was also determined for each neuron. The Watson-Williams *F*-test was used to examine whether different groups of neuron differed significantly in their mean angles of firing (Oriana). Significance for the Rayleigh and Watson-Williams tests was set at p < 0.05.

### Further Statistical Testing

The single-sample Kolmogorov-Smirnov test was used to judge whether noncircular data sets were normally distributed (p ≤ 0.05 to reject). Because some data sets were not normally distributed, we employed non-parametric statistical testing throughout (SigmaStat; Systat Software). The Mann-Whitney rank sum test was used for comparisons of unpaired data, with significance set at p < 0.05.

## Figures and Tables

**Table 1:** Distinct Local Axon Collaterals and Dendritic Architecture of GP-TI and GP-TA Neurons

Cell Type	Cell Number	Local Axon Collateral Length (mm)	Number of Varicosities in GPe	Density of Dendritic Spines (mm^−1^)
GP-TI	1	11.1	410	5.9
GP-TI	2	9.0	502	4.3
GP-TI	3	13.8	538	45.1
GP-TI	4	10.2	480	2.7
GP-TI	5	11.0	412	24.0
Mean for GP-TI neurons		11.0 ± 0.8	468 ± 25	16.4 ± 8.1
GP-TA	6	4.7	198	61.1
GP-TA	7	3.2	39	30.4
GP-TA	8	3.3	29	119.1
GP-TA	9	5.4	142	83.1
GP-TA	10	4.6	191	50.5
Mean for GP-TA neurons		4.2 ± 0.4∗	120 ± 36∗	68.8 ± 15.2∗

∗ Different as compared to GP-TI neurons; p < 0.02 Mann-Whitney rank sum tests. Data are means ± SEM.

## Supplemental Information

Document S1. Supplemental Experimental Procedures
