# Supplementary material for: Dichotomous Organization of the External Globus Pallidus
Source: Neuron. 2012 Jun 21;74(6):1075–86. doi: 10.1016/j.neuron.2012.04.027 (PMC3407962; doi:10.1016/j.neuron.2012.04.027)
Supplement: Document S1. Supplemental Experimental Procedures [file mmc1.pdf]

Neuron, Volume *74*
Supplemental Information

Dichotomous Organization of the External Globus Pallidus

Nicolas Mallet, Benjamin R. Micklem, Pablo Henny, Matthew T. Brown, Claire Williams, J. Paul Bolam, Kouichi C. Nakamura, and Peter J. Magill

Supplemental Experimental Procedures

Experimental procedures were carried out on adult male Sprague-Dawley rats (Charles River), and were conducted in accordance with the United Kingdom Animals (Scientific Procedures) Act, 1986.

6-hydroxydopamine lesions of dopamine neurons. Unilateral 6-hydroxydopamine (6-OHDA) lesions were induced in 190-305 g rats, as described previously (Mallet et al., 2008a; Mallet et al., 2008b). The neurotoxin 6-OHDA (hydrochloride salt; Sigma) was dissolved in 0.9% w/v NaCl solution containing 0.02% w/v ascorbate to a final concentration of 4 mg/ml. Twenty five minutes before the injection of 6-OHDA, all animals received desipramine (25 mg/kg, i.p.; Sigma) to minimize the uptake of 6-OHDA by noradrenergic neurons (Schwarting and Huston, 1996b). Anesthesia was induced and maintained with isoflurane (Schering-Plough) in O_2, and 3 μl of 6-OHDA solution was injected near the medial forebrain bundle (4.1 mm posterior and 1.2 mm lateral of Bregma, and 7.9 mm ventral to the dura [Paxinos and Watson, 1986]). Lesions were assessed 14 or 15 days after 6-OHDA injection by challenge with apomorphine (0.05 mg/kg, s.c.; Sigma; see Schwarting and Huston, 1996a), and were considered successful when animals made ≥80 net contraversive rotations in 20 min. Electrophysiological recordings were carried out in the GPe ipsilateral to 6-OHDA lesions in anesthetized rats 21-45 days after surgery.

In vivo electrophysiological recording and juxtacellular labeling of single neurons. Recording and labeling experiments were performed in 45 anesthetized 6-OHDA-lesioned rats (271-540 g at the time of recording). Briefly, anesthesia was induced with 4% v/v isoflurane in O_2, and maintained with urethane (1.3 g/kg, i.p.; ethyl carbamate, Sigma), and supplemental doses of ketamine (30 mg/kg, i.p.;

Willows Francis) and xylazine (3 mg/kg, i.p.; Bayer), as described previously (Mallet et al., 2008a; Mallet et al., 2008b). Wound margins were infiltrated with local anesthetic (0.5% w/v bupivacaine; Astra). Animals were then placed in a stereotaxic frame (Kopf). Body temperature was maintained at 37 ± 0.5°C by a homeothermic heating device (Harvard Apparatus). Electrocorticograms (ECoGs) and respiration rate were monitored constantly to ensure the animals' well being. The epidural ECoG was recorded above the frontal (somatic sensory-motor) cortex (4.0 mm anterior and 2.0 mm lateral of Bregma [Paxinos and Watson, 1986]), and was referenced against the ipsilateral cerebellar hemisphere (Mallet et al., 2008a). Raw ECoG was band-pass filtered (0.3-1,500 Hz, –3 dB limits) and amplified (2,000×; DPA-2FS filter/amplifier; npi electronic) before acquisition. Extracellular recordings of single-unit activity in the GPe were made using glass electrodes (11-29 MΩ *in situ*; tip diameter ~1.2 µm) containing 0.5 M NaCl solution and neurobiotin (1.5% w/v; Vector Laboratories). Electrodes were lowered into the brain under stereotaxic guidance and using a computer-controlled stepper motor (IVM-1000 or Burleigh/EXFO IW-711; both Scientifica), which allowed electrode placements to be made with submicron precision. Electrode signals were amplified (10×) through the bridge circuitry of an Axoprobe-1A amplifier (Molecular Devices), AC-coupled, amplified another 100×, and filtered at 300-5,000 Hz (DPA-2FS filter/amplifier). The ECoG and single-unit activity were each sampled at 17.9 kHz using a Power1401 Analog-Digital converter and a PC running Spike2 acquisition and analysis software (Cambridge Electronic Design). Following electrophysiological recordings, single neurons were juxtacellularly labeled with neurobiotin (Magill et al., 2001; Mallet et al., 2008a; Pinault, 1996). Briefly, positive current pulses (2-10 nA, 200 ms, 50% duty cycle) were applied until the single-unit activity became robustly entrained by the pulses. Single-unit entrainment resulted in just one neuron being labeled with neurobiotin. Seventy nine individual GPe neurons were juxtacellularly labeled in this study. Four to ten hours after labeling, animals were euthanized and transcardially perfused with 100 ml of 0.05 M phosphate-buffered saline, pH 7.4 (PBS), followed by 300 ml of 4% w/v paraformaldehyde in 0.1 M phosphate buffer, pH 7.4 (PB), and then left overnight in fixative at 4ºC. Brains used for electron microscopy were perfused with 4% paraformaldehyde and 0.01% v/v glutaraldehyde in PB.

Tissue processing for light microscopy. Parasagittal sections (50 µm) were cut from each brain using a vibrating microtome (VT1000S; Leica Microsystems), collected in series and washed in PBS. Free-floating sections were then incubated overnight at room temperature in 'Triton PBS' (PBS with 0.3% v/v Triton X-100 [Sigma]) containing Cy3-conjugated streptavidin (1:1,000 dilution; Life Technologies Corp.). Sections containing neurobiotin-labeled neuronal somata (those marked with Cy3) were then isolated for molecular characterization by indirect immunofluorescence. All identified GPe neurons were tested for expression of parvalbumin (PV), and some for choline acetyltransferase (ChAT) and/or preproenkephalin (PPE). Briefly, after 1-2 h of incubation in Triton PBS containing 10% v/v normal donkey serum (NDS; Jackson ImmunoResearch Laboratories), sections were incubated overnight at room temperature in Triton PBS containing 1% v/v NDS and one or more of the following primary antibodies: Guinea pig anti-PV (1:1,000 dilution; Synaptic Systems, 195004), goat anti-ChAT (1:500; Chemicon (Millipore), AB144P), rabbit anti-PPE (1:5,000; LifeSpan Biosciences, LS-C23084). To optimize immunolabeling for PPE, that is, to readily distinguish PPE+ GPe neurons from the high levels of PPE immunoreactivity in striatopallidal axons, we used a heat pre-treatment as a means of antigen retrieval (Jiao et al., 1999). Thus, before incubation in primary antibodies, sections were incubated in a 10 mM sodium citrate solution, pH 6.0, and then heated (80ºC for 6 h) in the same buffer. In some experiments (see Fig.6E,F), we also revealed neighboring GPe neurons by incubating sections in guinea pig anti-PV and mouse anti-human neuronal protein HuC/HuD ('HuCD'; 1:200; Life Technologies, A-21271). Separate experiments confirmed that PV+/PPE– and PV–/PPE+ cells also expressed HuCD (data not shown). After exposure to primary antibodies, sections were washed in PBS and incubated for 4-8 h at room temperature in Triton PBS containing fluorophore-conjugated secondary antibodies (all raised in donkey); AlexaFluor488 (1:500; Life Technologies), AMCA (1:250), or DyLight649 (1:500) (both Jackson ImmunoResearch). All secondary antibodies were highly cross-adsorbed by the manufacturers to reduce cross-species reactivity. After washing in PBS, sections were mounted in Vectashield (Vector) and viewed on confocal fluorescence microscopes (Zeiss LSM 510 or LSM 710). Neurochemical verification was performed by assessing single-plane confocal images acquired

using a constant 1.0 µm optical section (obtained by varying the pin hole size) and a 40× 1.3 NA or a 63× 1.4 NA oil-immersion objective lens. Each fluorescence channel was imaged in a separate track. Cy3 fluorescence was imaged with excitation from a Helium/Neon 543 nm laser, with emission restricted on the LSM 510 by a LP 560 filter (for the shorter wavelengths) and a NFT 635 VIS 2° dichroic (for longer wavelengths). Emission on the LSM 710 was restricted by selecting an emission range of 552-639 nm. AlexaFluor488 fluorescence was imaged with excitation from an Argon 488 nm laser, with emission restricted by either a BP 510-530 filter (on the LSM 510) or by setting emission at 493-542 nm (on the LSM 710). AMCA fluorescence was imaged on the LSM 710 with excitation from a diode 405 nm laser, and selecting emission at 409-485 nm. DyLight649 fluorescence was imaged with excitation from a Helium/Neon 633 nm laser, with emission restricted by either a LP 650 filter (LSM 510) or by setting emission at 637-757 nm (LSM 710). Absence of crosstalk between channels or ‘bleed through’ was assured for each fluorescence reaction. A neuron was classified as not expressing the tested molecular marker only when positive immunoreactivity could be observed in other cells on the same focal plane as the tested neuron. Digital images were cropped to regions of interest, with brightness and contrast adjusted when necessary, using Photoshop software (Creative Suite 3; Adobe Systems).

To visualize the somatodendritic and axonal architecture of identified neurons using brightfield microscopy, we then revealed the neurobiotin tracer with a permanent reaction product (Magill et al., 2001; Sadek et al., 2007). Tissue sections were incubated overnight at room temperature in Triton PBS containing Avidin-Biotinylated peroxidase Complex (ABC Elite, Vector), washed in PBS and then incubated in 0.05M Tris buffer (pH 8.0) containing 0.002% w/v hydrogen peroxide, 0.025% w/v 3,3'-diaminobenzidine tetrahydrochloride (DAB; Sigma) and 0.5% w/v nickel ammonium sulfate for ~15 min. Neurobiotin-filled neurons were thus intensely labeled with an insoluble, black/blue precipitate (Ni-DAB). If pallidal and striatal targets of Ni-DAB-labeled GPe neurons were not to be identified, sections were then washed, mounted on glass slides, dried overnight, treated in ethanol and then xylene, and protected with mounting medium (DePeX; Gurr) and a coverslip. When targets of GPe neurons were to be identified (see Figs.5 and 6), sections not

containing Ni-DAB-labeled somata were further processed by the ‘peroxidase-anti-peroxidase’ (PAP) method to reveal other neurons expressing PV, nitric oxide synthase (NOS) or ChAT (Bevan et al., 1998). Structures expressing PV were localized by overnight incubations in PBS containing, in order of application, rabbit anti-PV (1:1,000; Swant, PV25), goat anti-rabbit IgG (1:200, Dako), and rabbit PAP (1:750; Dako). After washing sections in PBS, immunoreactivity was then revealed by incubation in 0.05 M Tris buffer (pH 7.4) containing 0.01% w/v hydrogen peroxide and 0.05% w/v DAB for 10 min. The DAB reactions resulted in immunoreactive structures being labeled with a light-brown precipitate that was readily distinguished from the black/blue labeling of neurobiotin-containing neuronal structures. Immunoreactivity for NOS was revealed by incubations in mouse anti-NOS (1:500; Sigma, N2280), rabbit anti-mouse IgG (1:200; Dako), mouse PAP (1:500; Dako), and finally, the DAB solution detailed above. Immunoreactivity for ChAT was revealed by incubations in goat anti-ChAT (1:200), rabbit anti-goat IgG (1:100; Abcam), goat PAP (1:1,000; Jackson) and the DAB solution. Note that immunoreactivities for PV, NOS and ChAT were not revealed on the same tissue sections.

Digital reconstruction of the dendritic and axonal architecture of single neurons. Reconstructions were performed blind to electrophysiological phenotype. Five identified GP-TI neurons and five GP-TA neurons (cells #1-10; see Figs.3 and 4) were traced in three dimensions using Neurolucida software (MBF Bioscience) and a light microscope (Nikon Eclipse 80i) equipped with a motorized x-y-z stage and a Lucivid (MBF Bioscience) (Sadek et al., 2007). Digital reconstruction was only performed if just one neuron was juxtacellularly labeled in the brain. Boundaries of basal ganglia nuclei were drawn using a 10× objective, whereas Ni-DAB-labeled neuronal processes were drawn in fine detail using a 100× oil-immersion objective (1.4 NA; Plan Apo, Nikon). Locations of axonal boutons were recorded, and additional markers placed where boutons were apposing DAB-labeled somata or dendrites. After all sections had been drawn, reconstructions were spliced and corrected for shrinkage in the z-axis (Sadek et al., 2007). Morphometric analyses, including quantifications of dendritic lengths/branching, spine numbers/density, axon lengths, and numbers of boutons/appositions were carried out using Neurolucida Explorer software (MBF Bioscience). High resolution two-

dimensional vector files of projection views (medial or dorsal; see Figs.3 and 4) were created by printing from Neurolucida Explorer to a virtual PostScript printer, with further processing in Illustrator software (Adobe). Brightfield micrographs (see Figs.5 and 6) were taken with 60× or 100× oil-immersion objective lenses and a Retiga 2000R camera (QImaging), using the stack acquisition function of Neurolucida. These stacks were processed to extended-focus micrographs using Helicon Focus software (HeliconSoft).

Tissue processing for electron microscopy. Electron microscopy was carried out according to our standard protocols (Sadek et al., 2007), and was only performed if just one GP-TA neuron was juxtacellularly labeled in the brain. Briefly, 50 μm-thick parasagittal sections were first equilibrated overnight in a cryoprotectant solution (0.05 M PB containing 25% w/v sucrose and 10% v/v glycerol). Sections were then freeze-thawed (to enhance reagent penetration) by freezing in isopentane cooled in liquid nitrogen, followed by liquid nitrogen, and then thawing and washing in PBS. Neurobiotin was then revealed with a Ni-DAB reaction product as above (but without Triton detergent). Subsequently, sections were post-fixed in PB containing 1% w/v osmium tetroxide (Oxkem) for 25 min, dehydrated through a graded series of ethanol solutions (with 1% w/v uranyl acetate [TAAB Laboratories] added to the 70% ethanol solution), treated with propylene oxide (Sigma), and equilibrated in resin overnight (Durcupan ACM; Fluka). Sections were then mounted on glass slides, and cured at 60°C for 48 h. After examination in the light microscope, pieces of striatal tissue containing the axonal arborizations of GP-TA neurons were dissected out. Serial ultrathin sections (~50 nm) were cut on an ultramicrotome (EM UC6; Leica) and collected on single-slot copper grids coated with pioloform (Agar Scientific). Ultrathin sections were then contrasted with lead citrate (Sadek et al., 2007) for 2-3 min, and examined in a Philips CM100 electron microscope. Images from the electron microscope were digitally recorded using an UltraScan 1000 CCD digital camera (Gatan). For labeled axon terminals forming synapses, images of serial sections (up to 10) were recorded. The striatal structures postsynaptic to GPe axon terminals (i.e., dendritic shafts or spines) were characterized. Spines were identified on the basis of their emergence from a dendritic shaft, their relatively small size, the absence of mitochondria, and/or the presence of spine apparatus. When a labeled axon terminal made

synaptic contact with a dendritic shaft, we endeavored to verify whether the dendrite arose from a spiny projection neuron by looking for the emergence of spines in serial ultrathin sections. If spines could not be found, the cellular origin of the postsynaptic target was not assumed, and it was recorded as unidentified (although many such dendrites were likely derived from aspiny interneurons [Tepper and Bolam, 2004]). Electron micrographs were viewed and manipulated for contrast and brightness using ImageJ (NIH, http://rsbweb.nih.gov/ij/) and Photoshop (Adobe).

Electrophysiological data analysis. Epochs of robust cortical slow-wave activity or cortical activation were first visually inspected and selected for analysis (Mallet et al., 2008a; Mallet et al., 2008b). All analyses were performed on 100 s of data. The classification of GPe units as 'GP-TI' or 'GP-TA' was performed by computing the 'activity histogram' (custom scripts in MATLAB v7.3; Math Works) of single-unit activity with respect to the cortical slow (~1 Hz) oscillation (Mallet et al., 2008a). For statistical definition of temporal relationships between spiking and the cortical slow oscillation, we employed Pearson's χ^2 test (Excel; Microsoft Corp.) or, when relatively inactive neurons were tested, the non-parametric binomial test (SPSS; SPSS Inc.) (Mallet et al., 2008a). Significance for χ^2 and binomial tests was set at $P < 0.05$. Neurons firing at mean rates of <0.2 Hz were not statistically tested (see Results). The coefficient of variation of the interspike interval (CV_{isi}) was calculated as an indicator of firing regularity. Linear phase histograms were used to quantify the temporal relationships between the firing of identified GPe neurons and cortical beta oscillations (Mallet et al., 2008a). Modulations of unit activity in time with cortical beta oscillations were tested for significance using Rayleigh's Uniformity Test (Oriana; Kovach Computing). The null hypothesis for Rayleigh's test was that the spike data were distributed in a uniform manner. The mean angle of spike firing, with respect to the peaks of the cortical oscillation (defined as 0/360 degrees), was also determined for each neuron. The Watson-Williams *F*-test was used to examine whether different groups of neuron differed significantly in their mean angles of firing (Oriana). Significance for Rayleigh's and Watson-Williams tests was set at $P < 0.05$.

Further statistical testing. The single-sample Kolmogorov-Smirnov test was used to judge whether noncircular data sets were normally distributed ($P \leq 0.05$ to reject). Because some data sets were not

normally distributed, we employed non-parametric statistical testing throughout (SigmaStat; Systat Software). The Mann-Whitney rank sum test was used for comparisons of unpaired data, with significance set at $P < 0.05$.

Supplemental References

Paxinos, G., and Watson, C. (1986). The Rat Brain in Stereotaxic Coordinates, 2nd Edn (Sydney, Australia: Academic Press).
